# Measured Parental Weight Status and Familial Socio-Economic Status Correlates with Childhood Overweight and Obesity at Age 9

**DOI:** 10.1371/journal.pone.0043503

**Published:** 2012-08-17

**Authors:** Eimear Keane, Richard Layte, Janas Harrington, Patricia M. Kearney, Ivan J. Perry

**Affiliations:** 1 Department Epidemiology and Public Health, University College Cork, Cork, Ireland; 2 Economic and Social Research Institute, Sir John Rogerson’s Quay, Dublin, Ireland; Chancellor College, University of Malawi, Malawi

## Abstract

**Background:**

Parental obesity is a predominant risk factor for childhood obesity. Family factors including socio-economic status (SES) play a role in determining parent weight. It is essential to unpick how shared family factors impact on child weight. This study aims to investigate the association between measured parent weight status, familial socio-economic factors and the risk of childhood obesity at age 9.

**Methodology/Principal Findings:**

Cross sectional analysis of the first wave (2008) of the Growing Up in Ireland (GUI) study. GUI is a nationally representative study of 9-year-old children (N = 8,568). Schools were selected from the national total (response rate 82%) and age eligible children (response rate 57%) were invited to participate. Children and their parents had height and weight measurements taken using standard methods. Data were reweighted to account for the sampling design. Childhood overweight and obesity prevalence were calculated using International Obesity Taskforce definitions. Multinomial logistic regression examined the association between parent weight status, indicators of SES and child weight. Overall, 25% of children were either overweight (19.3%) or obese (6.6%). Parental obesity was a significant predictor of child obesity. Of children with normal weight parents, 14.4% were overweight or obese whereas 46.2% of children with obese parents were overweight or obese. Maternal education and household class were more consistently associated with a child being in a higher body mass index category than household income. Adjusted regression indicated that female gender, one parent family type, lower maternal education, lower household class and a heavier parent weight status significantly increased the odds of childhood obesity.

**Conclusions/Significance:**

Parental weight appears to be the most influential factor driving the childhood obesity epidemic in Ireland and is an independent predictor of child obesity across SES groups. Due to the high prevalence of obesity in parents and children, population based interventions are required.

## Introduction

The rising prevalence of childhood obesity is a major public health concern worldwide. Currently, one in four Irish children are either overweight or obese [1]. Parental obesity is well established as an important risk factor for childhood obesity [2]–[6]. Having an overweight parent doubles [7], [8] the risk of child obesity while obesity amongst both parents further increases the risk [4], [8], [9].

The relationship between parent and child weight is complex as it is a consequence of both shared genetic and environmental factors [10]–[13]. Socio-economic status (SES) is an important determinant of the shared family environment. Numerous studies have demonstrated an association between SES and obesity [14]. SES can influence lifestyle choices and behaviours, area of residence and food affordability, all of which are factors that have been shown to be associated with obesity [15]–[18].

The inverse association between SES and obesity in adults is well established [19]. However, evidence of a relationship between childhood obesity and SES remains equivocal [13], [20]–[23]. Variation in the types and definition of SES indicators used in studies may partly explain this. A review by Shrewsbury and Wardle [20] suggested that the association between child weight and SES is dependent on the type of SES indicator assessed. Parental education appeared to be most consistently associated with childhood obesity [20]. However, evidence of an association between household class and household income with child obesity remained less consistent [20].

As the prevalence of childhood overweight and obesity continues to increase, it is essential to unpick how shared family factors impact on child weight. Understanding the underlying pathways to childhood obesity will help in the development of effective policies and interventions against child obesity. This present research utilizes nationally representative data containing detailed information on three key indicators of SES as well as objective measures of parental weight status and this provides a unique opportunity to determine the effect of different family factors on childhood obesity. In this present paper we (1) estimate the prevalence of childhood overweight and obesity by measured parental weight status and a range of SES indicators and (2) investigate the association between parental weight status, familial socio-economic characteristics and the risk of childhood obesity at age 9.

## Methods

### Ethics Statement

Written informed consent was obtained from a parent/guardian and the study child prior to data collection commencing. Ethical approval was granted by the Research Ethics Committee (REC) of the Health Research Board based in Dublin, Ireland.

### Study Design and Sample

The study sample comprised of 8,568 nine-year old children who participated in the first wave (2007–8) of the Growing Up in Ireland (GUI) study [24]. GUI is a nationally representative cohort of 9 year old children residing in the Republic of Ireland. The sample was collected using a two-stage sampling method within the national school system. Eligible children were those who were born between the 1st November 1997 and the 31st October 1998. In the first stage, 1,105 primary schools from the national total of 3,200 were randomly selected using a probability proportionate to size (PPS) sampling method. In the second stage, a random sample of eligible children were selected from within each school. At the school level, a response rate of 82% was achieved, while at the household level (i.e. eligible child selected within the school) 57% of children and their parents participated in the study. The data was probability weighted prior to analysis to account for the complex sampling design. This involved the structural adjustment of the study sample to the population level whilst maintaining the case base of 8,568 children [25], [26].

### Procedures

Trained social interviewers conducted computer assisted personal interviews with the study child and both parents/guardians (where applicable) within the home. Parents nominated a primary caregiver (the parent who spent most time with the study child) who was the primary respondent. Mothers were the primary caregiver for 98% of the study children. Responses to sensitive questions were self-reported on a paper questionnaire.

### Anthropometric Measures

Anthropometric measurements were obtained during the household interview using validated methods [25]. The interviewers were responsible for height and weight measurements of each study child and each adult respondent. Height was recorded to the nearest millimetre using a Leicester portable height stick. Weight was recorded using a SECA 761 flat mechanic scales to the nearest 0.5 kilogram. Study children and their parents were asked to wear light clothing for the weight measurement. Children were classified as normal weight, overweight (a body mass index [BMI] of 19.46 for boys and 19.45 for girls) or obese (a BMI of 23.39 for boys and 23.46 for girls) using age and gender specific International Obesity Taskforce (IOTF) cut off points [27]. Measured parent BMI was classified according to the World Health Organization classifications as normal weight (<25 kg/m^2^), overweight (≥25 and <30 kg/m^2^) or obese (≥30 kg/m^2^) [28].

### Covariates

Parent reported variables were study child’s gender (male/female), family type (one parent/two parents), study child has siblings (yes/no), mother’s current age and SES indicators. Mother’s current age was categorized into four groups (<30, 30–39, 40–49, 50+). SES was assessed using three different indicators: household class, household income and mother’s highest level of education [24]. Mother’s highest level of education (as opposed to father’s highest level of education) was chosen as they tended to be the primary caregiver. The mother’s education variable was coded as follows: lower secondary education or less, higher secondary education, post-secondary education and third level education. Household class was measured using the Irish Central Statistics (CSO) Social Class Schema 1996 produced by aggregating occupations classified using the CSO’s Standard Classification of Occupations. For two parent families where both parents were economically active and were in different classes, the higher of the social classes was assigned to the family [24]. Net household income was self-reported. Net income was adjusted for household composition and size.

A separate variable was constructed for mother’s measured BMI classification and father’s measured BMI classification. Both variables were coded: normal weight, overweight, obese, missing. A combined single index variable for parent weight status was constructed by combining the mother’s and father’s measured BMI variables and was coded as: single parent/both parents normal weight (normal weight family), one parent overweight (in a two parent family), single parent/both parents overweight (overweight family), one parent obese (in a two parent family), single parent/both parents obese (obese family).

### Missing Data

No/low levels (<2%) of missing values were found within most of the covariates. However, where large levels of missing data were observed, methods of representing these values were incorporated into the analysis. Net household income had a high number (N = 626, 7.3%) of missing values. The continuous equivalised net income variable was imputed using the multiple imputation (MI) command in STATA. This variable was then re-coded and presented in quintiles. Measured height and/or weight data was missing for 5.2% of mothers and 6.4% of fathers (where present). Statistical tests suggested that the height and weight data were not missing at random so the data could not be imputed. In order to account for missing data, ‘missing data’ categories were generated for the mothers measured BMI and fathers measured BMI variables. Measured BMI data was available for 95% of the study children. This gave an effective case base of 8,136 children for analysis.

### Statistical Analysis

Analysis was completed in STATA 12 IC (StataCorp LP, USA). Probability weights were applied using survey data commands to account for the complex survey design. Prevalence estimates for normal weight, overweight and obese children were obtained. Unadjusted multinomial logistic regression was used to determine the risk of childhood overweight or obesity compared to normal weight according to parental weight status and familial SES factors. Forward stepwise multinomial logistic regression was conducted to assess the relationship between parent weight status, SES factors and childhood overweight and obesity. Non-significant variables based on the univariate regression (mother’s current age) were not included in the forward stepwise regression. Mother’s measured BMI and father’s measured BMI were not included during adjustment as they were combined to form the single index variable parent weight status. Each of the nested models presented in the results section were adjusted for socio demographic (study child’s gender, family type and study child has siblings) variables and SES indicators. Model 1 included the social demographic variables and household class; model 2 further adjusted for maternal education; model 3 was further adjusted for household income. The final model (model 4) was fully adjusted for study child’s gender, study child has siblings, household class, highest level of maternal education, household income and parent weight status.

## Results

Measured BMI data was available for 8,136 (95%) children. Overall, 74.1% (95% CI, 72.8–75.3) of children were a normal weight, 19.3% (95% CI, 18.2–20.5) were overweight and 6.6% (95% CI, 5.9–7.4) were obese. The prevalence of normal weight, overweight and obese children by parent weight status and by indicators of familial SES is shown in table 1.

**Table 1:** Prevalence of normal weight, overweight and obese 9 year old children by parental weight and family socio-economic status indicators.

			Prevalence N = 8136		
	Sample N = 8136	%	Normal weight N = 6120	Overweight N = 1545	Obese N = 471
***Gender***					
Boy	3958	51.3%	3101 (78.0%)	661 (16.6%)	196 (5.4%)
Girl	4178	48.7%	3019 (70.0%)	884 (22.2%)	275 (7.8%)
***Family type***					
Two parents	7215	82.2%	5474 (74.6%)	1352 (19.3%)	389 (6.1%)
One parent	921	17.8%	646 (71.6%)	193 (19.7%)	82 (8.7%)
***Has siblings***					
Yes	7340	89.73%	5569 (74.9%)	1346 (18.6%)	425 (6.5%)
No	626	8.8%	431 (66.0%)	156 (26.1%)	39 (7.9%)
***Mother’s age***					
<30	497	9.0%	350 (70.7%)	108 (21.3%)	39 (8.0%)
30–39	3107	41.34%	2303 (73.5%)	607 (19.5%)	195 (7.0%)
40–49	4271	46.82%	3282 (75.5%)	775 (18.7%)	214 (5.7%)
50+	219	2.9%	156 (70.6%)	47 (23.2%)	16 (6.2%)
***Household class***					
Professional workers	1114	8.25%	926 (81.9%)	165 (16.0%)	23 (2.1%)
Managerial and technical	3154	33.5%	2418 (76.6%)	594 (18.6%)	142 (4.7%)
Non-manual	1598	18.72%	1177 (72.8%)	316 (20.5%)	105 (6.8%)
Skilled manual	1137	16.63%	809 (71.6%)	234 (20.1%)	94 (8.3%)
Semi- skilled and unskilled	702	10.92%	479 (66.0%)	157 (23.0%)	66 (11.0%)
Unclassified class	431	11.98%	311 (74.4%)	79 (17.5%)	41 (8.1%)
***Equivalised household annual income (in quintiles)***					
Highest	2007	20.11%	1575 (76.9%)	363 (18.8%)	69 (4.3%)
4^th^	1734	20.1%	1301 (73.8%)	347 (19.9%)	86 (6.4%)
3^rd^	1513	20.2%	1120 (73.9%)	289 (19.9%)	104 (6.2%)
2^nd^	1300	19.95%	969 (72.6%)	241 (20.1%)	90 (7.3%)
Lowest	993	19.63%	718 (73.6%)	184 (17.4%)	91 (9.0%)
***Highest level of maternal education***					
Third level education	2103	16.87%	1694 (80.6%)	349 (16.6%)	60 (2.8%)
Post secondary education	2007	15.95%	1513 (75.2%)	384 (19.1%)	110 (5.7%)
Higher secondary education	2560	37.15%	1908 (74.6%)	493 (19.3%)	159 (6.1%)
Lower secondary education or less	1412	30.0%	968 (69.3%)	311 (21.3%)	133 (9.4%)
***Mother’s measured BMI classification***					
Normal	3836	47.16%	3207 (82.9%)	543 (14.6%)	86 (2.5%)
Overweight	2491	31.59%	1796 (70.7%)	523 (21.5%)	172 (7.9%)
Obese	1349	19.23%	804 (59.7%)	371 (27.2%)	174 (13.1%)
Missing	177	2.02%	135 (78.2%)	30 (14.7%)	12 (7.1%)
***Father’s measured BMI classification***					
Normal	1506	20.57%	1276 (83%)	192 (14.2%)	38 (2.8%)
Overweight	3439	46.96%	2680 (77.7%)	608 (17.7%)	151 (4.6%)
Obese	1713	25.57%	1107 (63.9%)	451 (25.5%)	155 (10.6%)
Missing data	452	6.91%	325 (67.7%)	88 (22%)	39 (10.3%)
***Parent Weight Status***					
Single parent/both parents normal weight	1271	18.86%	1104 (85.6%)	146 (12.6%)	21 (1.8%)
One overweight (2 parent family)	2139	26.69%	1803 (83.2%)	284 (14.1%)	52 (2.7%)
Single parent/both parents overweight	1340	18.75%	977 (72.4%)	276 (20.3%)	87 (7.3%)
One obese (2 parent family)	1922	25.78%	1317 (68.2%)	466 (23.9%)	139 (7.9%)
Single parent/both parents obese	575	9.92%	297 (53.8%)	180 (29.5%)	98 (16.7%)

In total, 30% of girls were overweight or obese compared with 22% of boys (p = 0.000). Within each of the SES indicators, there was an inverse relationship between SES and the prevalence of child overweight and obese. Those ranked lower within each of the socio-economic variables (household income p = 0.013, maternal education p = 0.000 & household class p = 0.000) were significantly more likely to be overweight or obese than those ranked at a higher position. A higher prevalence of overweight and obesity was found among children whose mothers were either overweight or obese compared with children whose fathers were overweight or obese (p = 0.000). Overall, 47.2% (95% CI, 45.7%–48.7%) of mothers were normal weight whilst 20.6% (95% CI, 19.4%–21.8%) of fathers were normal weight. Of children from two parent families, only 12% had 2 normal weight parents while 39.2% had at least one obese parent. In total, 11% (95% CI, 8.5%–14.1%) of children with 2 normal weight parents were overweight or obese. This increased to 24.7% (95% CI, 21.8%–28%) when one parent was obese and to 49.2% (95% CI, 43.3%–55.1%) when both parents were obese. Of children from single parent families, 49.2% (95% CI, 45.1%–53.3%) had a normal weight parent and 20% (95% CI, 16.7%–23.9%) had an obese parent. Overall, 18.1% (95% CI, 14.1%–23%) of children from single parent families with a normal weight parent were overweight or obese. This increased to 34.1% (95% CI, 27.7%–41.2%) when the parent was overweight and 41% (95% CI, 32%–50.6%) when the parent was obese.

Table 2 presents the results of the univariate multinomial logistic regression analyses. Univariate regression indicates that female gender, one parent family type, being an only child, lower household class, lower maternal education, lower household income and higher parental BMI (mother’s BMI, father’s BMI and parent weight status) were all associated with a child being in a higher BMI category. Having an overweight parent (within mother’s BMI, father’s BMI and the combined single index variable parent weight status) consistently increased the odds of childhood overweight and obesity. Parent weight status was most strongly associated with childhood overweight and obesity. The univariate regression also indicated that a lower household class and lower maternal education were associated with greater odds of childhood obesity than household income.

**Table 2:** Association between parental weight status, family socio-economic status indicators and the risk of child overweight and obesity.

	Overweight		Obese	
	OR (95% CI)	*P*	OR (95% CI)	*P*
***Gender***				
Boy	1		1	
Girl	1.49 (1.29–1.72)	0.000	1.61 (1.27–2.03)	0.000
***Family type***				
Two parent	1		1	
One parents	1.07 (0.87–1.31)	0.529	1.47 (1.09–2)	0.013
***Has siblings***				
Yes	1		1	
No	1.07 (1.01–1.14)	0.016	0.97 (0.86–1.1)	0.660
***Mother’s age***				
<30	1		1	
30–39	0.88 (0.65–1.19)	0.404	0.84 (0.54–1.31)	0.445
40–49	0.82 (0.62–1.1)	0.181	0.67 (0.44–1.03)	0.065
50+	1.09 (0.67–1.78)	0.731	0.78 (0.38–1.6)	0.5
***Household class***				
Professional workers	1		1	
Managerial & technical	1.25 (0.97–1.61)	0.088	2.4 (1.35 – 4.26)	0.003
Non-manual	1.44 (1.11–1.88)	0.006	3.61 (1.96 – 6.64)	0.000
Skilled manual	1.44 (1.09–1.9)	0.011	4.49 (2.43–8.32)	0.000
Semi- skilled &unskilled	1.79 (1.32–2.43)	0.000	6.45 (3.41–12.18)	0.000
Unclassified class	1.21 (0.84–1.74)	0.306	4.2 (2.13–8.3)	0.000
***Highest level of maternal education***				
Third level education	1		1	
Post secondary education	1.23 (1–1.51)	0.046	2.21 (1.42–3.43)	0.000
Higher secondary education	1.26 (1.04–1.52)	0.018	2.4 (1.6–3.6)	0.000
Lower secondary education or less	1.49 (1.21–1.84)	0.000	3.96 (2.66–5.89)	0.000
***Equivalised household annual income (in quintiles)***				
Highest	1		1	
4^th^	1.1 (0.9–1.34)	0.353	1.53 (1.02–2.29)	0.038
3^rd^	1.1 (0.89–1.36)	0.378	1.5 (1.02–2.2)	0.041
2^nd^	1.13 (0.91–1.4)	0.276	1.79 (1.19–2.68)	0.005
Lowest	0.96 (0.75–1.24)	0.769	2.18 (1.44–3.31)	0.000
***Mother’s measured BMI classification***				
Normal	1		1	
Overweight	1.73 (1.46–2.05)	0.000	3.65(2.64–5.06)	0.000
Obese	2.59 (2.12–3.16)	0.000	7.17 (5.13–10.03)	0.000
Missing data	1.07 (0.63–1.82)	0.799	2.98 (1.52–5.85)	0.002
***Father’s measured BMI classification***				
Normal	1		1	
Overweight	1.33 (1.07–1.65)	0.010	1.74 (1.13–2.69)	0.012
Obese	2.33 (1.86–2.93)	0.000	4.92 (3.2–7.57)	0.000
Missing data	1.89 (1.33–2.69)	0.000	4.51 (2.56–7.97)	0.000
***Parent Weight Status***				
Single parent/both parents normal weight	1		1	
One overweight (2 parent family)	1.16 (0.89–1.50)	0.275	1.54 (0.85–2.79)	0.157
Single parent/both parents overweight	1.91 (1.45–2.50)	0.000	4.74 (2.70–8.32)	0.000
One obese (2 parent family)	2.39 (1.84–3.1)	0.000	5.42 (3.15–9.32)	0.000
Single parent/both parents obese	3.73 (2.69–5.17)	0.000	14.53 (8.17–25.85)	0.000

Results of the forward stepwise multinomial logistic regression are presented table 3. The social demographic variables, female gender (p = 0.000) and one parent family type (p = 0.000) were significantly associated with childhood obesity. One parent family type was no longer significantly associated with childhood obesity when the SES indicators were added to the model (model 3: p = 0.173). When household income was added to model 3, household income was no longer significantly associated with the risk of a child being in a higher BMI category. However, the association between household class and maternal education with child BMI remained unchanged (when comparing model 3 to model 2). In the fully adjusted model (model 4), female gender, one parent family type, lower household class, lower maternal education and having overweight or obese parents significantly increased the risk of child obesity. Within model 4, children whose mothers were educated to less than a graduate level had at least double the odds of childhood obesity compared with those educated to a graduate level. A lower household class remained significantly associated with child obesity. Although not significant, lower levels of education and a lower household class were associated with a higher risk of childhood overweight. Parent weight status was most significantly associated with childhood overweight and obesity. Children with obese parents were at a significantly increased odds of overweight (OR 3.9, 95% CI, 2.8–5.6) when compared to children with normal weight parents. The odds of childhood obesity were 15.3 (95% CI, 8.4–27.7) when the single parent/both parents were obese. The odds of childhood obesity increased by nearly 3 fold when the single parent/both parents were obese compared to the single parent/both parents being overweight.

**Table 3:** Forward stepwise multinomial logistic regression.

					Model 1																Model 2						Model 3						Model 4			
					Overweight							Obese									Overweight				Obese		Overweight		Obese				Overweight		Obese	
					OR (95% CI)				*P*			OR (95% CI)						*P*			OR (95% CI)		*P*		OR (95% CI)	*P*	OR (95% CI)	*P*		OR (95% CI)	*P*		OR (95% CI)	*P*	OR (95% CI)	*P*
***Gender***																																				
*** Boy***						1					1										1				1		1			1		1			1	
*** Girl***						1.47 (1.28–1.7)		0.000			1.52 (1.21–1.91)						0.000				1.46 (1.26–1.68)			0.000	1.48 (1.18–1.86)	0.001	1.44 (1.24–1.67)	0.000		1.52 (1.19–1.94)	0.001	1.45 (1.24–1.70)		0.000	1.52 (1.15–2.0)	0.003
***Family type***																																				
*** Two***					1						1									1					1		1			1		1			1	
*** One***					1.1 (0.86–1.4)				0.464		1.34 (0.94–1.9)						0.108			1.15 (0.9–1.47)				0.277	1.33 (0.93–1.91)	0.119	1.23 (0.95–1.59)	0.112		1.3 (0.89–1.89)	0.173	1.47 (1.09–1.97)		0.011	1.83 (1.17–2.87)	0.009
***Siblings***																																				
*** Yes***					1						1									1					1		1			1		1			1	
*** No***					1.07 (1.01–1.13)				0.031		0.95 (0.83–1.08)					0.417				1.07 (1.01–1.13)				0.033	0.95 (0.83–1.09)	0.449	1.06 (1.0–1.14)	0.055		0.95 (0.83–1.10)	0.505	1.07 (0.99–1.14)		0.080	0.95 (0.83–1.09)	0.493
***Household class***																																				
** Professional workers**				1							1									1					1		1			1		1			1	
** Managerial &technical**				1.23 (0.95–1.6)					0.114		2.33 (1.31–4.16)				0.004					1.17 (0.90–1.52)				0.249	2.7 (1.61–4.52)	0.000	1.15 (0.88–1.5)	0.304		2.55 (1.51–4.31)	0.000	1.17 (0.88–1.57)		0.278	2.89 (1.55–5.39)	0.001
** Non-manual**				1.40 (1.07–1.83)					0.016		3.39 (1.81–6.33)				0.000					1.26 (0.94–1.69)				0.121	3.34 (1.93–5.78)	0.000	1.21 (0.89–1.65)	0.213		3.17 (1.81–5.55)	0.000	1.25 (0.9–1.75)		0.180	3.26 (1.7–6.25)	0.000
** Skilled manual**				1.40 (1.05–1.86)					0.021		4.37 (2.36–8.10)				0.000					1.27 (0.93–1.72)				0.127	3.98 (2.27–6.99)	0.000	1.26 (0.91–1.75)	0.156		3.78 (2.12–6.73)	0.000	1.32 (0.93–1.88)		0.120	4.0 (2.01–7.81)	0.000
** Semi-skilled &unskilled**				1.69 (1.24–2.31)					0.001		5.93 (3.1–11.35)				0.000					1.50 (1.07–2.10)				0.018	5.01 (2.76–9.09)	0.000	1.50 (1.06–2.14)	0.024		4.40 (2.36–8.20)	0.000	1.43 (0.98–2.1)		0.066	4.75 (2.29–9.86)	0.000
** Unclassified class**				1.11 (0.74–1.67)					0.602		3.26 (1.51–7.05)				0.003					0.97 (0.63–1.48)				0.888	2.75 (1.37–5.54)	0.005	0.97 (0.62–1.53)	0.898		2.62 (1.27–5.37)	0.009	0.94 (0.56–1.57)		0.805	2.13 (0.96–4.76)	0.064
***Highest level of maternal education***																																				
** Third level**			–								–									1					1		1			1		1			1	
** Post secondary**																				1.18 (0.95–1.46)				0.131	1.91 (1.21–3.01)	0.005	1.2 (0.95–1.51)	0.120		1.89 (1.18–3.03)	0.008	1.18 (0.92–1.52)		0.202	2.29 (1.47–3.55)	0.000
** Higher secondary**																				1.16 (0.94–1.43)				0.162	1.9 (1.23–2.94)	0.004	1.17 (0.94–1.46)	0.156		1.81 (1.14–2.88)	0.012	1.11 (0.87–1.41)		0.422	2.05 (1.35–3.11)	0.001
** Lower secondary/less**																				1.33 (1.05–1.70)				0.018	2.79 (1.77–4.39)	0.000	1.41 (1.08–1.83)	0.010		2.79 (1.72–4.53)	0.000	1.22 (0.91–1.64)		0.117	2.7 (1.72–4.23)	0.000
***Equivalised household annual income (in quintiles)***																																				
** Highest**		–									–											–			–		1			1		1			1	
** 4^th^**																											1.01 (0.82–1.24)	0.933		1.16 (0.77–1.75)	0.488	0.96 (0.77–1.2)		0.713	1.19 (0.76–1.86)	0.459
** 3^rd^**																											0.96 (0.77–1.21)	0.744		0.94 (0.63–1.41)	0.780	0.93 (0.73–1.2)		0.589	0.96 (0.61–1.52)	0.873
** 2^nd^**																											0.96 (0.75–1.21)	0.703		1.02 (0.66–1.58)	0.927	0.91 (0.71–1.18)		0.487	0.96 (0.59–1.55)	0.864
** Lowest**																											0.81 (0.60–1.08)	0.146		1.11 (0.69–1.78)	0.656	0.75 (0.55–1.04)		0.083	1.04 (0.61–1.75)	0.895
***Parent weight status***																																				
** Single parent/both parents normal weight**	–									–												–			–		–			–		1			1	
** One overweight (2 parent family)**																																1.32 (0.99–1.77)		0.058	2.16 (1.16–4.18)	0.022
** Single parent/both parents overweight**																																2.08 (1.56–2.79)		0.000	5.36 (2.95–9.72)	0.000
** One obese (2 parent family)**																																2.66 (2.0–3.55)		0.000	6.88 (3.76–12.61)	0.000
** Single parent/both parents obese**																																3.94 (2.78–5.58)		0.000	15.28 (8.44–27.65)	0.000

## Discussion

Using nationally representative data this present study aimed to assess the association between measured parent weight status, familial SES factors and the risk of childhood obesity. This research has resulted in two principal findings. Firstly, parent weight status appears to be the most significant independent predictor of childhood obesity in Ireland. Children from families with overweight or obese parents were at a significantly higher risk of obesity than children with normal weight parents. Secondly, household class and maternal education are better predictors of childhood obesity than household income.

Only 18.9% of children were from families (either single parent or two parent families) with normal weight parents. Having normal weight parents appears to have a protective effect against the risk of childhood obesity. Only 14.4% of children from such families were overweight or obese whereas 46.2% of children with obese parents were overweight or obese. After adjustment for household socio-economic characteristics, children from obese parent families remained at greater than 15 (95% CI, 8.44–27.65) times the odds of obesity when compared to children from families with normal weight parents. This suggests that SES alone cannot explain the association between parent obesity and child obesity. SES indicators appear to only capture some shared familial environmental factors which result in weight gain. We suggest that these results highlight that the shared family environment is a multi-dimensional contributor to the obesity epidemic with both genetic and environmental origins.

Within this present study, children who were more deprived were at a higher risk of overweight and obesity, which is similar to results found in adults [19]. Children from one parent families were found to be at significantly higher odds of overweight and obesity than children from two parent families. Some research suggests that one parent families may have greater levels of social deprivation and this may play a role in explaining this [29]. However, our results indicate that parental weight was more predictive overweight and obesity in children from single parent families than SES. There was an inverse association between household class and maternal education with childhood obesity. The association between household class and childhood obesity was more graded. Within the final adjusted model, children from a lower household class were at higher odds of obesity than children with lesser educated mothers. Research indicates that parental education is the SES indicator most consistently associated with childhood obesity [20], [21]. This may be because maternal education is a more stable indicator of SES over time than household income or household class. Maternal education is likely to influence factors including literacy as well as knowledge of healthy versus unhealthy behaviours which impact on weight status [14], [30]. As a higher level of education appears protective against child obesity, this suggests that education may be crucial in tackling the obesity epidemic. Overall, variations in odds of obesity by each indicator of SES suggest that household class, household income and maternal education may all influence different behaviours and choices that impact weight gain. Further research is required to fully understand how each SES characteristic predicts behaviours which result in weight gain. In addition, efforts are necessary to standardise SES indicators and definitions used across studies.

In this study SES indicators do not explain all the association between parent and child weight. Therefore, other causal pathways for childhood obesity need to be considered. Research from other studies of childhood obesity indicate that the weight status of parents from 2 parent families may interact [4], [31]. Mechanisms resulting in a positive energy balance in both parents appear to be more predictive of childhood obesity than such mechanisms in one parent. In this current research having 2 obese parents compared with one obese parent resulted in a 2 fold increase in the odds of childhood obesity.

A study by Wardle et al. [32] compared food, physical activity and lifestyle patterns in children from lean and obese families. This study found that children from obese families had higher preferences for fatty foods and sedentary activities and a lower preference for fruit and vegetable consumption. Such food and physical activity patterns may have a negative impact on energy balance resulting in weight gain. Such diet and activity patterns may potentially explain the lack of significance for household income in this present study. Parent weight status may be a better predictor of food types purchased rather than income or other measures of household SES. More affluent families with obese parents may have a preference for energy dense food regardless of income available to spend on good quality foods. Grunert at al. [33] suggest that habitual behaviour is difficult to change even if an individual is aware of the negative consequences of their behaviours. Grunert et al. suggest that obese individuals have a greater response to external cues (sight, smell) for food intake whilst normal weight individuals respond to internal cues (hungry). Children may acquire habitual behaviours and responses to dietary and physical activity patterns from that of their parents. Another possible explanation is that genotypes including the FTO gene which impacts appetite may influence control over food intake and choices resulting in children from obese families having a greater predisposition for obesity [34]–[37].

Similar to other findings [38], [39], maternal obesity was more predictive of a child being in a higher BMI category than paternal obesity. There are a number of possible explanations for this. Mothers were nominated as the primary caregiver (the person who spent most time with the study child) for 98% of children who took part in this study. This indicates that children spend more time in their mother’s environment and thus may acquire more behaviour’s from their mother. A study by Hannon et al. [40] found that the eating habits of the family food preparer, 84% of whom were mothers, predicted the eating habits of their child. Birth factors including the role of the intra-uterine environment on subsequent risk of childhood obesity is a second possible explanation [41], [42].

### Strengths and Limitations

GUI is a large and nationally representative sample. The sample equates to approximately one in seven of all births in Ireland in 1997. The results of the study are applicable at a population level as a result of applying the sampling weights. All objective BMI measurements were measured by trained professionals using validated techniques. The study contains information on three indicators of SES (household class, equivalised household income and maternal highest level of education). Imputing the household income variable decreased the amount of missing data.

However, there are several limitations to the study. There was a relatively low response rate at the household level (57%). The data have been weighed to adjust for the sampling strategy and response rate. However, there may be residual response bias. Of the children with measured BMI, there was missing values for BMI for 2% of mothers and 6.9% of fathers. Data was also missing for income for 7.3% of the households. While the missing data imputation procedure has enhanced the study power, it would have been preferable not to have missing data on this key variable. The data analysed in this present research is cross-sectional. Therefore, a causal relationship cannot be inferred though as the children are only 9 years old it is likely that parental and SES factors partly predicted the onset of obesity.

### Conclusions

Parent weight status is a significant predictor of childhood obesity. Children from lower household class families and those with lesser educated mothers were at an increased risk of childhood obesity. Early intervention is required to tackle the problem of childhood obesity. It may be suggested to target interventions at families where parents are overweight or obese. However, we must consider that in the current study, this includes the majority (81%) of families. Thus, the findings highlight the need for broadly based population level interventions targeting the social, economic and cultural dimensions of overweight and obesity. Further research is needed to assess how behaviours that affect energy balance vary between families with normal weight parents versus families with obese parents.
